# Impact of vaccine pause due to Thrombosis with thrombocytopenia syndrome (TTS) following vaccination with the Ad26.COV2.S vaccine manufactured by Janssen/Johnson & Johnson on vaccine hesitancy and acceptance among the unvaccinated population

**DOI:** 10.1371/journal.pone.0274443

**Published:** 2022-10-11

**Authors:** Daniel A. Salmon, Holly B. Schuh, Rikki H. Sargent, Alexis Konja, Steven A. Harvey, Shaelyn Laurie, Brandy S. Mai, Leo F. Weakland, James V. Lavery, Walter A. Orenstein, Robert F. Breiman

**Affiliations:** 1 Institute for Vaccine Safety, Johns Hopkins Bloomberg School of Public Health, Baltimore, Maryland, United States of America; 2 Department of International Health, Johns Hopkins Bloomberg School of Public Health, Baltimore, Maryland, United States of America; 3 Department of Health, Behavior and Society, Johns Hopkins Bloomberg School of Public Health, Baltimore, Maryland, United States of America; 4 Department of Epidemiology, Johns Hopkins Bloomberg School of Public Health, Baltimore, Maryland, United States of America; 5 RIWI Corp., Toronto, Ontario, Canada; 6 Global Health Crisis Coordination Center (GHC3), Atlanta, Georgia, United States of America; 7 Hubert Department of Global Health, Rollins School of Public Health, Emory University, Atlanta, Georgia, United States of America; 8 Center for Ethics, Emory University, Atlanta, Georgia, United States of America; 9 School of Medicine, Emory University, Atlanta, Georgia, United States of America; SUNY Downstate Health Sciences University, UNITED STATES

## Abstract

**Background:**

In response to reports of thrombosis with thrombocytopenia syndrome (TTS) post-vaccination, the Johnson & Johnson (J&J) vaccine was paused and then restarted in April 2021. Our objective was to assess whether this pause adversely impacted vaccine confidence.

**Methods:**

Two large internet-based surveys were conducted in the US among adults to measure knowledge, attitudes and perceptions of the J&J vaccine pause and rates of vaccine hesitancy among unvaccinated persons before, during and after the pause.

**Results:**

Among 66% of respondents aware of the pause, 44% identified blood clots as the reason for the pause without prompting. The impact of the pause on vaccine behavior among unvaccinated persons and perception of the vaccine safety system was mixed and modified by trust in the public health authorities. Those who were less willing to get vaccinated because of the pause were less inclined for all vaccines, not only the J&J product. Moreover, a notable proportion (22.1%) of the small number of persons (n = 30) vaccinated with the J&J vaccine after the pause reported not receiving information about the risk of TTS. The proportion of unvaccinated persons who were hesitant was increasing before and during the pause and then leveled off after the pause.

**Conclusions:**

The J&J vaccine pause is unlikely to be a major barrier to vaccine uptake. Public attitudes about vaccines may be more resilient than appreciated, especially when safety issues are investigated with transparent communication. This paper has important implications for messaging and program administration with future vaccine-specific adverse events. Efforts may be warranted to ensure all persons being offered the J&J vaccine are made aware of the risk of TTS.

## Introduction

Several vaccines received emergency use authorizations in the United States (US) beginning in December 2020, less than a year after China announced the first cases of COVID-19. Vaccine development was accelerated by decades of technologic advances coupled with massive government support. Severe acute respiratory syndrome coronavirus 2 (SARS-CoV-2) vaccines hold great promise to end the pandemic. Less than 6 months after vaccine introduction, supply was outpacing demand in the US despite not achieving sufficient vaccine coverage to achieve herd immunity in all communities.

Attitudes toward COVID-19 disease and vaccines as well as vaccine coverage vary by age, ethnicity, education, political party affiliation, and region [1]. Concerns that the vaccines were rushed and not adequately studied for safety have been a major contributor to suboptimal vaccine acceptance [1, 2]. Adverse events following immunization, whether actually caused by vaccine or coincidental, can increase doubt among people who otherwise lean toward immunization and confirm doubts for those who lean against [2].

On April 13, the Centers for Disease Control and Prevention (CDC) and Food and Drug Administration (FDA) paused the use of the Janssen Ad26.COV2.S vaccine manufactured by Johnson & Johnson (J&J) in the US in response to reports of Thrombosis with thrombocytopenia syndrome (TTS) post-vaccination, primarily among women under 50 years of age. On April 23, following thorough review of available data, the agencies lifted the pause and use of J&J vaccine resumed.

While some criticized the pause as a distraction from the push for increased coverage, the pause and rigorous assessment demonstrated that vaccine safety was monitored, and signals were promptly investigated. With the objective of informing policy decisions around future safety issues, we set out to characterize the impact of the J&J vaccine pause on COVID-19 vaccine hesitancy in the general, unvaccinated population as well as age-, sex-, race-, and region-based subpopulations. We provide results from two large internet-based surveys to assess the impact of TTS on COVID-19 vaccine hesitancy.

## Methods

### Survey administration

For our primary analysis, we conducted and pooled data from two Web-based US surveys using RIWI’s (Real-Time Interactive World-Wide Intelligence) patented Random Domain Intercept Technology (RDIT) (S1 and S2 Appendices and https://www.protocols.io/view/impact-of-vaccine-pause-due-to-thrombosis-with-thr-cdbzs2p6). The vaccination intent survey was administered March 30—May 1, 2021. The rapid response (J&J) survey was administered April 30—May 13, 2021. We conducted the rapid response survey while implementing the vaccination intention survey and designed it specifically to continue capturing COVID-19 vaccine attitudes, beliefs, and behaviors while collecting additional cross-sectional information on attitudes and beliefs unique to the J&J pause event and immediately following its occurrence. We conducted a secondary descriptive analysis using data from the second survey on attitudes and beliefs unique to the J&J pause event to provide further context to the main analysis measuring the change in overall-, age-, sex-, race-, and region-specific vaccine hesitancy over time.

When internet users happen upon one of the hundreds of thousands of rotating domains that RIWI owns or controls (“dead links” that do not go to a functional website), these randomly engaged users are filtered through a series of proprietary algorithms to ensure that they are human respondents and subsequently invited to participate in a survey [3–6]. Respondents access surveys on all Web-enabled devices with internet access. Since all Web users have random chances of coming across RIWI surveys, respondents are geographically representative of internet users. The vaccination intent survey exposed over a million Web users to the survey landing page; of those, 78,697 opted-in to participate in the survey (6.8% opt-in rate). Over 600,000 Web users were exposed to the rapid response (J&J) survey, and 34,284 opted-in to participate (5.3% opt-in rate). The methodology collects the age, gender, and geo-location of respondents but no personally identifiable information. Respondents are informed that responses are fully anonymous and secure. No enticements are used; participants can exit at any point [7, 8]. Consent is implied by participant participation. This project was determined by Emory and JHU to be public health practice and not human subjects research.

### Survey content

Both the vaccine intent and rapid response J&J surveys asked respondents if they had been vaccinated with at least one dose of a COVID-19 vaccine and, if not, asked them to select among scales of intention to get vaccinated. In addition, a variety of demographic and personal characteristics were collected along with questions about trusted sources of information and respondents’ reasons to get or not get vaccinated. In addition to assessing vaccination status and intention, we asked respondents to the J&J rapid response survey about their trust in the CDC and FDA, if they had heard about the J&J vaccine safety issue that caused the pause and, if so, what they had heard (free text), and if what they heard influenced their attitudes. Respondents were provided a brief description of the safety issue. Unvaccinated respondents were asked if the information about the J&J vaccine issue impacted their willingness to get the J&J vaccine or the available mRNA COVID-19 vaccines. All respondents were asked if the information impacted their trust in the vaccine safety monitoring systems.

### Outcome of interest

The primary outcome variable of interest, denoted as Y_i_ (*i* = 30 March 2021–13 May 2021), was the daily proportion of unvaccinated survey participants who were hesitant or against getting vaccinated during three periods relative to the vaccination pause using the Johnson and Johnson (J&J) vaccine: 1) pre-pause (30 March– 12 April; 14 days); 2) during pause (13–23 April; 11 days); 3) after pause (24 April– 13 May; 21 days). The outcome variable was derived from a categorical variable based on a question asked to unvaccinated survey participants about their attitude toward COVID-19. When asked “what will you do when it is your turn to get the COVID-19 vaccine at no cost?”, participants selected from a list of categories: “I will…”:

- “…definitely get it as soon as I can”,
- “…likely get it as soon as I can”,
- “… likely get it but not right away”,
- “…likely not get vaccinated”, or
- “…definitely not get vaccinated”.

The dichotomization of the categorical term was based on the vaccine hesitancy elements described in the present body of literature, mainly confidence or acceptance of the COVID-19 vaccines as well as an element of time (e.g. delay) when getting vaccinated [9–11]. Those hesitant (delayed or unlikely) or against getting vaccinated were those who answered “likely but not right away”, “unlikely will”, and “definitely will not”. All proportions were weighted using survey design weights. We required at least 100 participants for any given day when calculating overall and stratified population proportions, and all proportions were weighted using design-based weights.

### Covariates of interest

The main exposure of interest is the J&J pause with intervention time points defined as: 1) start of the J&J pause and 2) end of the J&J pause. We constructed dummy variables to indicate the different segmented periods (pre-intervention periods_1-2_ versus post-intervention periods_1-2_ using 0 and 1 codes, respectively). We looked at age and sex differences, stratifying by women (risk of TTS was reported among women) under the age of 50 years, women over the age of 50 years, and men. We explored race/ethnicity and COVID-19 vaccination coverage-based regional differences (25 June 2021 vaccination rank-based tertiles were determined to create regions indicating low, medium, and high relative coverage) by looking at daily proportions across sub-groups.

### Statistical analysis

Analyses were performed using the two-sided significance level of 0.05. Weights were based on age and gender using the most recent national census data available (US Census Bureau 2021 projections) and were generated post-stratification using a raking algorithm. Chi-square tests were used to inspect the differences in the distribution of survey participant demographic characteristics, and p-values were calculated with a Rao-Scott second-order correction. Covariates missingness was evaluated and if greater than 5%, multiple imputation using 25 replications was conducted where missingness did not exceed ~30%. Data were combined from both surveys to ensure we had at least 100 participants for any given day when calculating overall and stratified population proportions, and all proportions were weighted [13].

Interrupted time series analysis estimated daily proportions of COVID-19 unvaccinated individuals who were COVID-19 vaccine hesitant or refusing pre-, during-, and post- J&J vaccine pause, using multiple segmented linear regression models [12]. Both surveys were required to carry out segmented regression. The level and slope parameters define the intercept and slope, respectively, for successive segments of the time series [11]. The level parameters were defined as the expected or average proportion of hesitant unvaccinated individuals at a specific time interval, and the slope parameters indicate the change in the proportion over a single unit of time (per day). Serial autocorrelation of error terms were tested using residual plots and by calculating the Durbin-Watson statistic using an estimate of close to 2.00 to rule out important autocorrelation [13]. The postestimation command *estat hettest* was used to test for heteroskedasticity to verify constant variance of all plots, [12, 14, 15] and *rreg* to look at possible exclusion of potential outliers. For the final model, autocorrelation was adjusted for by estimating the autocorrelation parameter using the command *prais* and including it in the model (Durbin-Watson statistics before adjustment (2.56); after adjustment: 2.09) [12, 14]. Weighted-based chi-square estimates were used to inspect differences in distributions of age, sex, race/ethnicity, and geographic region across vaccination intention categories during the entire study period. Data were analyzed using Stata (version 17, 2-core) for Mac OS [12].

Stratified analyses on race/ethnicity and regions were conducted based on COVID-19 vaccination coverage [16] (high, middle, and low coverage tertiles), and a combination of age and gender (women less than 50 years of age, women 50 years of age and older, and men). Models were constructed and checked using the same methods described above. Cross-model comparisons testing predictions and marginal effects were conducted using seemingly unrelated estimation (*suest* and the *test* commands for Wald test) and a robust variance estimator to adjust for duplication of daily observations in the stack dataset [17, 18].

A secondary analysis, designed to provide context for the trend in COVID-19 vaccine hesitant/refusing attitudes among the unvaccinated using a descriptive analysis of knowledge, attitudes, and beliefs about the pause was conducted with data from the rapid response J&J survey. We used the same methods for these descriptive statistics as those for the descriptive portion of the primary interrupted time series analysis as described above.

## Results

### Primary analysis

#### Descriptive analysis

The proportion of unvaccinated individuals among all survey participants (n = 89,083) decreased from 68.0% to 64.1% to 61.1% during the pre-pause, during-pause, and post-pause time periods. Among the unvaccinated individuals (n = 56,193), distributions of age (p<0.01), sex (p<0.01), race/ethnicity (p<0.01), and region (p<0.01) differed significantly by vaccine hesitancy (Table 1). By survey (vaccine intent versus rapid response), statistically significant differences were not found by age (p = 0.30) or sex (p = 0.44) but did exist by race/ethnicity (p<0.01) and coverage-based region quintiles (p<0.01) (S1 Table). There were sex-, race/ethnicity-, and region-based differences by survey time period (each p<0.01) but no difference found by age (p = 0.07) (S2 Table).

**Table 1:** Sociodemographic characteristics by vaccine hesitancy status (hesitant/against versus not) (versus not) among unvaccinated respondents (n = 56,193, unweighted), unweighted/weighted.

All	Total	Vaccine hesitancy/resistance, unweighted		Total	Vaccine hesitancy/resistance, weighted		P-value
		No	Yes		No	Yes	
	N = 56193	N = 19494	N = 36699	N = 54727	N = 19102	N = 35625	
Age (years)							<0.01
18–29	19,656 (35.0%)	6,793 (34.8%)	12,863 (35.1%)	12144 (22.2)	4169 (21.8)	7976 (22.4)	
30–49	19,056 (33.9%)	6,511 (33.4%)	12,545 (34.2%)	19176 (22.2)	6518 (34.1)	12658 (35.5)	
50+	17,481 (31.1%)	6,190 (31.8%)	11,291 (30.8%)	23406 (35)	8415 (44.1)	14991 (42.1)	
Female	24,290 (43.2%)	8,069 (41.4%)	16,221 (44.2%)	28233 (51.6)	9486 (49.7)	18747 (52.6)	<0.01
Race or ethnicity							<0.01
White	16,691 (43.3%)	5,273 (39.8%)	11,418 (45.2%)	16876 (45.3)	5249 (40.7)	11627 (47.7)	
Black	5,607 (14.6%)	1,815 (13.7%)	3,792 (15.0%)	5114 (13.7)	1768 (13.7)	3346 (13.7)	
Hispanic / LatinX	4,943 (12.8%)	2,090 (15.8%)	2,853 (11.3%)	4381 (11.8)	1891 (14.7)	2490 (10.2)	
Asian	3,462 (9.0%)	1,550 (11.7%)	1,912 (7.6%)	3109 (8.3)	1381 (10.7)	1727 (7.1)	
AI/AN	3,264 (8.5%)	1,160 (8.8%)	2,104 (8.3%)	3499 (9.4)	1293 (10)	2206 (9.1)	
Other	4,538 (11.8%)	1,358 (10.3%)	3,180 (12.6%)	4301 (11.5)	1321 (10.2)	2980 (12.2)	
Educational attainment							0.10
High school	3,707 (38.3%)	1,355 (39.8%)	2,352 (37.5%)	3489 (35.8)	1261 (37.5)	2228 (34.8)	
Technical / vocational training	1,642 (17.0%)	554 (16.3%)	1,088 (17.4%)	1763 (18.1)	578 (17.2)	1185 (18.5)	
College degree	2,914 (30.1%)	1,039 (30.5%)	1,875 (29.9%)	3050 (31.3)	1032 (30.7)	2018 (31.6)	
Masters degree	1,409 (14.6%)	459 (13.5%)	950 (15.2%)	1455 (14.9)	491 (14.6)	964 (15.1)	
Urban / rural							
Rural	2,004 (20.3%)	481 (13.9%)	1,523 (23.9%)	2113 (21.3)	492 (14.3)	1620 (24.9)	
Town / village	2,406 (24.4%)	887 (25.6%)	1,519 (23.8%)	2394 (24.1)	873 (25.4)	1521 (23.4)	
Suburb	2,980 (30.2%)	1,141 (32.9%)	1,839 (28.8%)	3031 (30.5)	1126 (32.8)	1905 (29.3)	
Large city	2,465 (25.0%)	962 (27.7%)	1,503 (23.5%)	2397 (24.1)	940 (27.4)	1458 (22.4)	
Politics							<0.01
Democrat	2,092 (21.5%)	1,101 (32.2%)	991 (15.7%)	2067 (21.1)	1115 (33)	952 (14.8)	
Republican	2,678 (27.5%)	648 (18.9%)	2,030 (32.1%)	2914 (29.7)	689 (20.4)	2224 (34.5)	
Independent	4,974 (51.0%)	1,671 (48.9%)	3,303 (52.2%)	4833 (49.2)	1571 (46.5)	3262 (50.7)	
Household income							<0.01
<$20000	2,900 (30.5%)	1,101 (32.8%)	1,799 (29.3%)	2773 (29)	1056 (31.8)	1718 (27.4)	
$20000-$50000	2,324 (24.5%)	868 (25.8%)	1,456 (23.7%)	2384 (24.9)	865 (26.1)	1519 (24.3)	
$50001-$75000	1,511 (15.9%)	528 (15.7%)	983 (16.0%)	1578 (16.5)	533 (16.1)	1045 (16.7)	
$75001-$125000	1,273 (13.4%)	429 (12.8%)	844 (13.7%)	1356 (14.2)	436 (13.2)	920 (14.7)	
>$125000	1,491 (15.7%)	432 (12.9%)	1,059 (17.2%)	1486 (15.5)	428 (12.9)	1058 (16.9)	
Survey time							<0.01
Pre-J&J pause, <13Apr	12,597 (22.4%)	4,700 (24.1%)	7,897 (21.5%)	12224 (22.3)	4629 (24.2)	7595 (21.3)	
During J&J pause, 13-22Apr	10,180 (18.1%)	3,355 (17.2%)	6,825 (18.6%)	9870 (18)	3285 (17.2)	6586 (18.5)	
After J&J pause, > = 23Apr	33,416 (59.5%)	11,439 (58.7%)	21,977 (59.9%)	32632 (59.6)	11188 (58.6)	21444 (60.2)	
Region, by coverage							<0.01
Group A—VT, MA, CT, RI, ME, NH, NJ, MD, WA, NM	5,021 (10.7%)	1,922 (12.3%)	3,099 (10.0%)	4634 (10.5)	1790 (12.2)	2844 (9.7)	
Group B–OR, HI, CO, NY, DC, VA, MN, CA, PA, DE	13,956 (29.9%)	5,200 (33.2%)	8,756 (28.2%)	12545 (28.5)	4634 (31.5)	7911 (27.1)	
Group C–WI, NE, IA, MI, SD, FL, IL, OH, NC, KY, MT	11,260 (24.1%)	3,554 (22.7%)	7,706 (24.8%)	10818 (24.6)	3434 (23.3)	7385 (25.3)	
Group D–AL, IN, KS, NV, TX, AZ, MO, SC, ND, OK	10,739 (23.0%)	3,410 (21.8%)	7,329 (23.6%)	10566 (24)	3372 (22.9)	7194 (24.6)	
Group E–WV, UT, GA, ID, TN, LA, AR, WY, AL, MS	5,747 (12.3%)	1,585 (10.1%)	4,162 (13.4%)	5381 (12.2)	1484 (10.1)	3897 (13.3)	
Concern of vaccine safety, yes	28,463 (80.6%)	8,865 (72.1%)	19,598 (85.1%)	27927 (81.7)	8762 (73)	19165 (86.4)	<0.01

Missingness, unweighted: race/ethnicity (31.5%); Educational status (82.8%); Urban/rural (82.5%); Political leaning (82.7%); Household income (83.1%); Region (16.9%)

#### Impact of pause on vaccination intent among the unvaccinated

**Fig 1:**
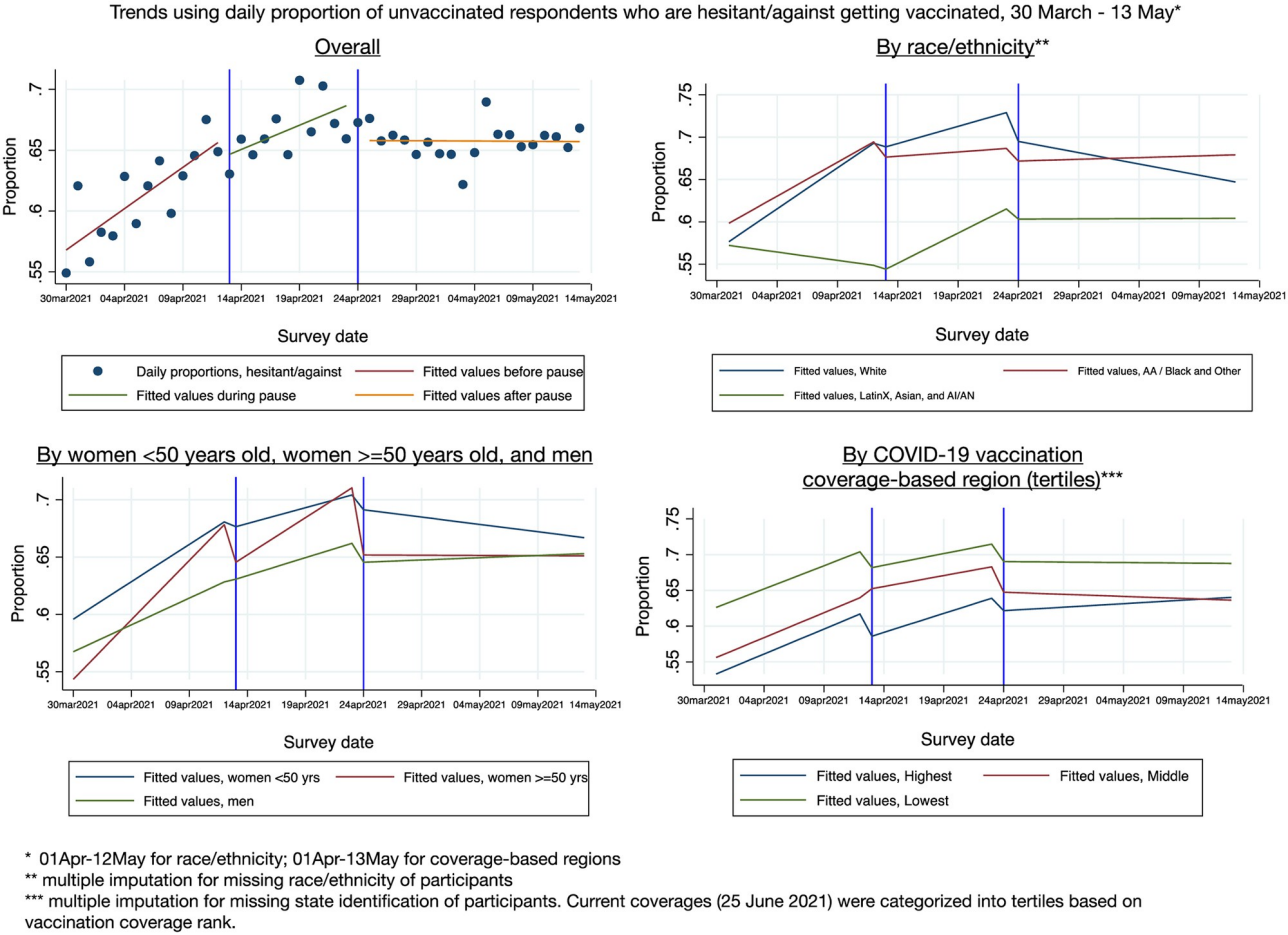
Trends in proportion of unvaccinated individuals (n = 56,193) with hesitant/against COVID-19 vaccination attitudes over three time periods (pre-, during-, and post-J&J pause), overall and stratified by race/ethnicity, women <50 years of age versus women > = 50 years of age and men, and COVID-19 coverage-based regions.

The overall trend in daily proportions of hesitant individuals was increasing prior to the pause (0.7% per day, 95% CI 0.5–0.9% per day, p<0.01) and continued to increase during the pause (0.4% per day, 95% CI 0.1–0.7% per day, p = 0.01) (Fig 1 and Table 2); the pre-pause and during-pause trend difference was not statistically significant (p = 0.10). The change in the daily proportions of hesitant individuals stabilized following the end of the pause and showed no daily increase or decrease (0% per day, range -0.1%-0.1%, p = 0.94). The trend change following the end of the pause is significant (-0.4% per day, 95% CI -0.7%-0.1%, p = 0.010).

**Table 2:** Overall trend in COVID-19 vaccine hesitancy/refusal among unvaccinated individuals before, during and after J&J pause from segmented regression model [17, 18].

Variable	Coefficient	95% CI	p-value
Pre-Pause trend in hesitancy	0.007	[0.005, 0.009]	<0.001
Pause trend in hesitancy	0.004	[0.001, 0.007]	0.007
Change in hesitancy trend after start of pause	-0.003	[-0.006, -0.001]	0.104
Post-Pause trend in hesitancy	0	[-0.001, 0.001]	0.940
Change in hesitancy trend after end of pause	-0.004	[-0.007, -0.001]	0.010

Note: Weighted analysis.

*** <0.01;

** <0.05;

* <0.01.

Adjusted for autocorrelation. N = 45 timepoints (no evidence of outliers). A positive coefficient means daily trend (slope) or the difference/change between the slopes of two time periods is increasing/increased and negative coefficient means the daily trend (slope) or the difference/change between the slopes of two time periods is decreasing/reduced.

Definition, Hesitancy: unvaccinated persons who reported “will likely get vaccinated, but not right away” or “will likely not get vaccinated” or “will definitely not get vaccinated”

Increasing daily proportions of vaccine hesitancy before and during the pause and the tempered, level trend (about 0%) after the pause were not different when comparing women younger than 50 years to women 50 years and older (p = 0.52) and women younger than 50 years to men (p = 0.49, see Fig 1).

Associations between vaccine hesitancy and the pause were consistent among White, African American/Black, and Other races (p = 0.12) where there was an increase in hesitancy leading up to the pause and during the pause that leveled off after the pause (Fig 1). However, among Latinx, Asian and American Indian or Alaskan Native (AI/AN) groups, which showed different trends in hesitancy compared to the White racial group as well as the African American/Black and Other racial group (p<0.01 for both comparisons), there was a decreasing trend in hesitancy prior to the pause, which reversed direction and hesitancy increased during the pause, and then daily proportions of hesitancy recovered and leveled off after the pause.

No significant differences in trends between high-, middle-, and low- COVID-19 coverage-based regions (p = 0.960) were observed. All three regions showed significant trends before the start of the pause exhibiting 1.0% (p = 0.01), 0.6% (p<0.01), and 0.8% (p<0.01) daily increases in the high, middle, and low coverage regions, respectively. No other time period trends or changes in the trends were found to be significant.

### Secondary analysis

#### Knowledge, attitudes, and beliefs

Among the 34,284 adults who were rapid response J&J survey respondents, the majority (66%) were aware of the pause and blood clot formation was most commonly identified as the reason for the pause without prompting (41%). Of those citing clotting, only 12% related it to occurring primarily in women. White respondents were less likely than other races to be unaware of the pause (S3 Table).

Among unvaccinated respondents, 48.3% said that the information we provided about the pause made them less willing to get vaccinated with the J&J vaccine; 43.1% were less willing to get mRNA vaccines. Willingness to get vaccinated was modified by trust in CDC. Among those who did not trust CDC, 63.8% were less willing to get vaccinated with a different COVID-19 vaccine (Pfizer or Moderna) because of the pause compared with 22.4% of persons who trusted CDC. Similarly, 44.4% of persons trusting CDC were more likely to be vaccinated with a different COVID-19 vaccine (Pfizer or Moderna) because of the pause compared with 12.7% of persons who did not trust CDC. Decreased willingness to receive the J&J vaccine was slightly more evident among those ≥50 years of age (50.7%), 18–25 years (45.3%) and 30–49 years (47.4%; p = 0.05), and affected women slightly more than men (50.3% versus 46.3%; p = 0.03). Rural and Republican respondents were more likely to report the information about the pause made them less likely to be vaccinated compared with other respondents (S3 Table). The information we provided about the pause had no impact (40.1%) or increased trust (33.9%) in the vaccine safety monitoring system for the majority of respondents. The impact of information about the pause on trust in the safety system was also modified by trust in the CDC. Among respondents trusting the CDC, 46.6% reported the pause increased trust in the safety system, compared with 19.1% of persons who did not trust CDC. Similarly, 51.6% of persons not trusting CDC reported the information about the pause made them less likely to trust the safety system, compared with 13.3% of persons who trusted CDC. Of the persons previously vaccinated, 5.7% felt worse about their decision to get vaccinated after reading the information, and 30.7% felt better.

The majority of respondents who received the J&J vaccine since the pause (78.0%) reported that they discussed information about the J&J safety issue and/or received written information about the J&J safety issue when they were vaccinated. A substantial proportion of these respondents (22.1%) received no discussion or written materials. A minority of unvaccinated respondents (14.1%) expressed a preference for the J&J vaccine and 31.3% would get it if it was the only vaccine available for them. Nearly all these factors varied by race and political affiliation (S3 Table) and many also varied by gender, age, income, and region (full survey in S2 Appendix).

## Discussion

We found that vaccine hesitancy generally increased from before and continuing through the J&J vaccination pause associated with the J&J vaccine adverse event but that related hesitancy leveled off after J&J immunization resumed, except for the subpopulation of Latinx, Asian, and AI/AN participants. Despite hesitancy leveling off, the end of the pause did not lead to a reduction in hesitancy. Important for messaging and program administration with future vaccine-specific adverse events, the associated concerns were related to all COVID-19 vaccines not just the J&J product, suggesting that adverse events in one vaccine can diminish trust in all. Nonetheless, perhaps demonstrating the value of transparent communication, the pause increased confidence in the vaccine safety system.

Even though TTS associated with this pause affected primarily younger women, the impact on daily trends in attitudes and intentions were not associated with gender or age suggesting that SARS CoV2 vaccine confidence is fragile across demographics. While there were differences in the impact of the pause on SARS CoV2 vaccine hesitancy by race and ethnicity, these were modest.

There are several important lessons from these findings. For the short term, the J&J vaccine pause is unlikely to be a major barrier to vaccine uptake for most unvaccinated persons. However, for a subset of the population, the pause may have reinforced existing concerns that the vaccines were developed too quickly and that additional safety problems may arise as the vaccines are used more broadly. Those who were already concerned about the safety of COVID-19 vaccines, particularly those who did not trust CDC, may have felt the pause validated their concerns. The net impact on vaccine acceptance was small. Reframing the pause as an example of the vaccine safety system functioning well may be useful in this subpopulation. In the long-term, public attitudes about vaccines may be more resilient than appreciated especially when safety issues are investigated with transparent communications to the public, even if doing so requires a pause in vaccination. This is the first study we are aware of that has been able to assess the impact of a vaccine safety issue on vaccine attitudes and intentions in real time. Retrospective review of the impact of vaccine safety scares are prone to many methodological challenges, typically including ecological rather than individual-level data [19].

There are several limitations to this study. The RIWI technology has many strengths but may be prone to non-response bias as all surveys. Although non-response bias is unknown, it is possible to assess trends in retention throughout a survey, which could yield further insights on the population of interest. While the methodology allows for an opt-in sample that is geographically representative of the population, it is possible for the sample to not be representative on other factors. To address this, we applied weights on age and gender in accordance with the United States Census. Of note, in our current data collection period (June 30—July 26, 2021), 72% of respondents (18 years of age and older) indicated that they have received at least one dose of the vaccine, which closely aligns with a contemporaneous CDC estimate of 68% [20]. All data including vaccine acceptance are self-reported. We used simple approaches to measuring some constructs, such as trust in FDA and CDC, whereas a longer survey could have used a more robust approach [13]. We found that among the 30 respondents who were vaccinated since the pause was lifted, 22.1% were not made aware of the risk of TTS which is concerning given the severity of TTS and the availability of alternative vaccines. However, these are self-reported data and the sample size is very small. In follow-up survey conducted immediately following this rapid survey (unpublished), we identified 1,845 persons who reported receiving the J&J vaccine after the pause; 17.5% reported no information about the risk of TTS was provided. Finally, we were not able to account for other factors that might impact vaccine hesitancy during this time period; however, this was the dominant story about the vaccine in the media and most people were aware of it.

Our study aligns with literature on the complexity of vaccine hesitancy. The end of a public vaccine safety event such as the J&J pause may have a tempering effect on the increasing proportion of vaccine hesitant attitudes among the remaining unvaccinated individuals over time—providing assurance that vaccine safety systems protect the public. Our study reinforces the importance of trust in public health authorities in response to a vaccine safety problem. Future research should focus on how trust in public health authorities can be improved and maintained. Public health authorities should be reassured that they can respond to a vaccine safety issue with transparency. A substantial proportion of the rapid response J&J survey respondents reported that they were not made aware of the risk of TTS at the time they were offered the J&J vaccine, though respondents may have under-reported or not recalled such warnings. While direct observation of the vaccination process would reduce this potential bias, efforts to ensure all persons being offered the J&J vaccine are made aware of the risk of TTS is warranted.

## Supporting information

S1 Table Sociodemographic characteristics by survey (Vaccination intention versus Rapid Response, J&J survey) (n = 54,727), weighted. (DOCX)

S2 Table Sociodemographic characteristics by survey time period (pre-, during-, or post- JJ pause) among unvaccinated respondents (n = 54,727), weighted. (DOCX)

S3 Table Rapid response J&J survey COVID-19 vaccine-related knowledge, attitudes, and beliefs by demographics, weighted. (DOCX)

S1 Appendix Data description. (XLS)

S2 Appendix Survey data. (DTA)

S1 Survey (DOCX)

S2 Survey (DOCX)
