# Supplementary material for: Impact of vaccine pause due to Thrombosis with thrombocytopenia syndrome (TTS) following vaccination with the Ad26.COV2.S vaccine manufactured by Janssen/Johnson & Johnson on vaccine hesitancy and acceptance among the unvaccinated population
Source: PLoS One. 2022 Oct 11;17(10):e0274443. doi: 10.1371/journal.pone.0274443 (PMC9553048; doi:10.1371/journal.pone.0274443)
Supplement: S1 Table — (DOCX) [file pone.0274443.s001.docx]

| Supplemental Table 1. Sociodemographic characteristics by survey (Vaccination intention versus Rapid Response, J&J survey) (n=54,727), weighted | | | | |
| --- | --- | --- | --- | --- |
|  | Total | Vaccine intention (baseline) | Rapid Response (J&J) | P-value |
|  | N=54727 | N=34194 | N=20533 |  |
| Age (years) |  |  |  | 0.30 |
| 18-29 | 12144 (22.2) | 7660 (22.4) | 4485 (0) |  |
| 30-49 | 19176 (35) | 11966 (0) | 7211 (0) |  |
| 50+ | 23406 (42.8) | 14569 (0) | 8837 (0) |  |
| Gender |  |  |  | 0.44 |
| Male | 26494 (48.4) | 26494 (48.5) | 28233 (48.2) |  |
| Female | 28233 (51.6) | 26494 (51.5) | 54727 (51.8) |  |
| Race or ethnicity |  |  |  | <0.01 |
| White | 16876 (45.3) | 15315 (44.8) | 1562 (50.6) |  |
| Black | 5114 (13.7) | 4740 (13.9) | 375 (12.1) |  |
| Hispanic / LatinX | 4381 (11.8) | 4029 (11.8) | 352 (11.4) |  |
| Asian | 3109 (8.3) | 2903 (8.5) | 205 (6.7) |  |
| AI/AN | 3499 (9.4) | 3294 (9.6) | 205 (6.7) |  |
| Other | 4301 (11.5) | 3914 (11.4) | 387 (12.5) |  |
| Educational attainment |  |  |  | 0.79 |
| High school | 3489 (35.8) | 2440 (35.6) | 1049 (36.2) |  |
| Technical / vocational training | 1763 (18.1) | 1251 (18.2) | 512 (17.7) |  |
| College degree | 3050 (31.3) | 2157 (31.4) | 893 (30.8) |  |
| Masters degree | 1455 (14.9) | 1013 (14.8) | 442 (15.3) |  |
| Urban / rural |  |  |  |  |
| Rural | 2113 (21.3) | 1504 (21.8) | 609 (20) |  |
| Town / village | 2394 (24.1) | 1654 (24) | 739 (24.3) |  |
| Suburb | 3031 (30.5) | 2089 (30.3) | 941 (30.9) |  |
| Large city | 2397 (24.1) | 1639 (23.8) | 759 (24.9) |  |
| Politics |  |  |  | <0.01 |
| Democrat | 2067 (21.1) | 1594 (23.2) | 473 (16) |  |
| Republican | 2914 (29.7) | 2106 (30.7) | 808 (27.4) |  |
| Independent | 4833 (49.2) | 3163 (46.1) | 1670 (56.6) |  |
| Household income |  |  |  | <0.01 |
| <$20000 | 2773 (29) | 1923 (28.3) | 850 (30.6) |  |
| $20000-$50000 | 2384 (24.9) | 1741 (25.6) | 643 (23.2) |  |
| $50001-$75000 | 1578 (16.5) | 1082 (15.9) | 496 (17.9) |  |
| $75001-$125000 | 1356 (14.2) | 1018 (15) | 339 (12.2) |  |
| >$125000 | 1486 (15.5) | 1037 (15.2) | 449 (16.2) |  |
| Survey time |  |  |  | <0.01 |
| Pre-J&J pause, <13Apr | 12224 (22.3) | 12224 (35.7) | 0 (0) |  |
| During J&J pause, 13-22Apr | 9870 (18) | 9870 (28.9) | 0 (0) |  |
| After J&J pause, >=23Apr | 32632 (59.6) | 12100 (35.4) | 20533 (100) |  |
| Region, by coverage |  |  |  | <0.01 |
| Group A | 4634 (10.5) | 3194 (10.8) | 1441 (10) |  |
| Group B | 12545 (28.5) | 8384 (28.4) | 4160 (28.8) |  |
| Group C | 10818 (24.6) | 7160 (24.3) | 3658 (25.3) |  |
| Group D | 10566 (24) | 6916 (23.5) | 3650 (25.2) |  |
| Group E | 5381 (12.2) | 3825 (13) | 1556 (10.8) |  |
| Vaccine safety |  |  |  |  |
| No | 6267 (18.3) | 6267 (18.3) | 6267 (18.3) |  |
| Yes | 27927 (81.7) | 27927 (81.7) | 27927 (81.7) |  |
| Vaccine hesitancy/resistance |  |  |  | 0.26 |
| No | 19102 (34.9) | 12001 (35.1) | 7101 (34.6) |  |
| Yes | 35625 (65.1) | 22194 (64.9) | 13431 (65.4) |  |
