# Supplementary material for: Impact of vaccine pause due to Thrombosis with thrombocytopenia syndrome (TTS) following vaccination with the Ad26.COV2.S vaccine manufactured by Janssen/Johnson & Johnson on vaccine hesitancy and acceptance among the unvaccinated population
Source: PLoS One. 2022 Oct 11;17(10):e0274443. doi: 10.1371/journal.pone.0274443 (PMC9553048; doi:10.1371/journal.pone.0274443)
Supplement: S2 Table — (DOCX) [file pone.0274443.s002.docx]

| Supplemental Table 2. Sociodemographic characteristics by survey time period (pre-, during-, or post- JJ pause) among unvaccinated respondents (n=54,727), weighted | | | | | |
| --- | --- | --- | --- | --- | --- |
|  | Total | Before J&J pause | During J&J pause | After J&J pause | P-value |
|  | N=54727 | N=12224 | N=9870 | N=32632 |  |
| Age (years) |  |  |  |  | 0.07 |
| 18-29 | 12144 (22.2) | 2766 (22.6) | 2172 (22) | 7206 (22.1) |  |
| 30-49 | 19176 (22.2) | 4246 (34.7) | 3572 (36.2) | 11358 (34.8) |  |
| 50+ | 23406 (35) | 5212 (42.6) | 4126 (41.8) | 14068 (43.1) |  |
| Gender |  |  |  |  | <0.01 |
| Male | 26494 (48.4) | 5755 (47.1) | 4811 (48.7) | 15929 (48.8) |  |
| Female | 28233 (51.6) | 6470 (52.9) | 5059 (51.3) | 16704 (51.2) |  |
| Race or ethnicity | |  |  |  | <0.01 |
| White | 16876 (45.3) | 5748 (47) | 4658 (47.2) | 6470 (42.6) |  |
| Black | 5114 (13.7) | 1675 (13.7) | 1421 (14.4) | 2018 (13.3) |  |
| Hispanic / LatinX | 4381 (11.8) | 1459 (11.9) | 1181 (12) | 1741 (11.5) |  |
| Asian | 3109 (8.3) | 932 (7.6) | 726 (7.4) | 1451 (9.6) |  |
| AI/AN | 3499 (9.4) | 1085 (8.9) | 833 (8.4) | 1581 (10.4) |  |
| Other | 4301 (11.5) | 1325 (10.8) | 1051 (10.6) | 1925 (12.7) |  |
| Educational attainment | |  |  |  | <0.01 |
| High school | 3489 (35.8) | 930 (35) | 794 (36.4) | 1765 (35.9) |  |
| Technical / vocational training | 1763 (18.1) | 461 (17.4) | 424 (19.5) | 878 (17.8) |  |
| College degree | 3050 (31.3) | 851 (32.1) | 648 (29.7) | 1551 (31.5) |  |
| Masters degree | 1455 (14.9) | 411 (15.5) | 315 (14.4) | 729 (14.8) |  |
| Urban / rural |  |  |  |  | 0.55 |
| Rural | 2113 (21.3) | 581 (21.7) | 494 (22.6) | 1037 (20.4) |  |
| Town / village | 2394 (24.1) | 643 (24.1) | 520 (23.8) | 1230 (24.2) |  |
| Suburb | 3031 (30.5) | 813 (30.4) | 665 (30.5) | 1553 (30.6) |  |
| Large city | 2397 (24.1) | 635 (23.8) | 504 (23.1) | 1258 (24.8) |  |
| Politics |  |  |  |  | <0.01 |
| Democrat | 2067 (21.1) | 672 (25.4) | 487 (22.3) | 908 (18.2) |  |
| Republican | 2914 (29.7) | 810 (30.6) | 692 (31.7) | 1412 (28.3) |  |
| Independent | 4833 (49.2) | 1167 (44) | 1005 (46) | 2661 (53.4) |  |
| Household income | |  |  |  | 0.05 |
| <$20000 | 2773 (29) | 732 (27.7) | 597 (27.6) | 1445 (30.2) |  |
| $20000-$50000 | 2384 (24.9) | 687 (26.1) | 561 (26) | 1136 (23.8) |  |
| $50001-$75000 | 1578 (16.5) | 419 (15.9) | 349 (16.2) | 810 (16.9) |  |
| $75001-$125000 | 1356 (14.2) | 408 (15.5) | 317 (14.7) | 632 (13.2) |  |
| >$125000 | 1486 (15.5) | 392 (14.9) | 334 (15.5) | 759 (15.9) |  |
| Region, by coverage | |  |  |  | <0.01 |
| Group A | 4634 (10.5) | 1236 (11.6) | 976 (11.1) | 2423 (9.9) |  |
| Group B | 12545 (28.5) | 3004 (28.1) | 2442 (27.7) | 7099 (29) |  |
| Group C | 10818 (24.6) | 2694 (25.2) | 2126 (24.1) | 5999 (24.5) |  |
| Group D | 10566 (24) | 2382 (22.3) | 2015 (22.9) | 6170 (25.2) |  |
| Group E | 5381 (12.2) | 1372 (12.8) | 1247 (14.2) | 2761 (11.3) |  |
| Vaccine safety | |  |  |  | <0.01 |
| No | 6267 (18.3) | 2586 (21.2) | 1801 (18.2) | 1881 (15.5) |  |
| Yes | 27927 (81.7) | 9639 (78.8) | 8069 (81.8) | 10219 (84.5) |  |
