# Supplementary material for: Impact of vaccine pause due to Thrombosis with thrombocytopenia syndrome (TTS) following vaccination with the Ad26.COV2.S vaccine manufactured by Janssen/Johnson & Johnson on vaccine hesitancy and acceptance among the unvaccinated population
Source: PLoS One. 2022 Oct 11;17(10):e0274443. doi: 10.1371/journal.pone.0274443 (PMC9553048; doi:10.1371/journal.pone.0274443)
Supplement: S3 Table — (DOCX) [file pone.0274443.s003.docx]

| Supplemental Table 3. Rapid response J&J survey COVID-19 vaccine-related knowledge, attitudes, and beliefs by demographics, weighted Note. All respondents were eligible to see survey questions designated with an "a" superscript. Only vaccinated respondents were eligible to see survey questions designated with a "b" superscript. Only unvaccinated respondents were eligible to see survey questions with a "c" superscript. Additional display logic was implemented as appropriate. P-values represent statistical significance of second-order Rao-Scott chi-square tests. All data come from the rapid response J&J survey with one exception—data for the item “when you were vaccinated, was any information about this safety issue with the J&J vaccine provided?” are preliminary results from a later survey, at which point a larger eligible sample size could be reached. | | | | | | | | | | | | | | | | | | | | | | | | | | | | | | | | | | | | | |
| --- | --- | --- | --- | --- | --- | --- | --- | --- | --- | --- | --- | --- | --- | --- | --- | --- | --- | --- | --- | --- | --- | --- | --- | --- | --- | --- | --- | --- | --- | --- | --- | --- | --- | --- | --- | --- | --- |
|  | **Overall** | |  | **Race** | | | | | | | | | | | | | |  | **Urban/Rural** | | | | | | |  | **Political Affiliation** | | | | | | | | | | |
|  |  |  |  | **White** | | **Black** | | **Hispanic, LatinX** | | **Asian** | | | **American Indian, Alaskan Native** | | **Other** | | ***P*** |  | **Rural Area** | | **Town/ Village or Suburb** | | **Large City** | | ***P*** |  | **Democrat/ Independent Lean**  **Democrat** | | | **Republican/ Independent Lean Republican** | | | **Independent** | | | ***P*** | |
| **Have you heard about any safety issues with the Johnson & Johnson (J&J) vaccine? ^a^** | | | | | | | | | | | | | | | | | | | | | | | | | | | | | | | | | | | | | |
| No | 6678 | (33.7%) |  | 887 | (24.4%) | 266 | (39.1%) | 254 | (35.7%) | 153 | (39.5%) | 147 | | (45.1%) | 251 | (40.1%) | <0.001 |  | 302 | (28.8%) | 1093 | (30.0%) | 532 | (33.0%) | 0.053 |  | 645 | (28.1%) | 579 | | (28.9%) | 627 | | (34.7%) | <0.001 | |  |
| Yes | 13151 | (66.3%) |  | 2748 | (75.6%) | 415 | (60.9%) | 458 | (64.3%) | 235 | (60.5%) | 180 | | (54.9%) | 376 | (59.9%) |  |  | 745 | (71.2%) | 2553 | (70.0%) | 1080 | (67.0%) |  |  | 1648 | (71.9%) | 1424 | | (71.1%) | 1182 | | (65.3%) |  | |  |
| **How does this information [on the safety issue with the J&J vaccine] impact your willingness to get the J&J vaccine? ^c^** | | | | | | | | | | | | | | | | | | | | | | | | | | | | | | | | | | | | | |
| Less Willing | 2138 | (48.3%) |  | 792 | (50.7%) | 185 | (49.4%) | 157 | (44.6%) | 86 | (42.0%) | 107 | | (52.0%) | 219 | (56.4%) | <0.001 |  | 365 | (60.0%) | 787 | (46.8%) | 366 | (48.3%) | <0.001 |  | 315 | (42.5%) | 662 | | (56.8%) | 499 | | (47.8%) | <0.001 | |  |
| Neutral | 1358 | (30.7%) |  | 593 | (37.9%) | 108 | (28.8%) | 107 | (30.3%) | 53 | (26.0%) | 55 | | (26.8%) | 102 | (26.4%) |  |  | 177 | (29.0%) | 606 | (36.1%) | 229 | (30.2%) |  |  | 227 | (30.6%) | 354 | | (30.4%) | 395 | | (37.8%) |  | |  |
| More Willing | 928 | (21.0%) |  | 178 | (11.4%) | 82 | (21.8%) | 89 | (25.2%) | 66 | (32.0%) | 44 | | (21.2%) | 67 | (17.2%) |  |  | 67 | (11.0%) | 287 | (17.1%) | 163 | (21.5%) |  |  | 200 | (27.0%) | 149 | | (12.8%) | 150 | | (14.4%) |  | |  |
| **How does this information [on the safety issue with the J&J vaccine] impact your willingness to get a different COVID-19 vaccine (Pfizer or Moderna)? ^c^** | | | | | | | | | | | | | | | | | | | | | | | | | | | | | | | | | | | | | |
| Less Willing | 1782 | (43.1%) |  | 731 | (46.8%) | 158 | (42.1%) | 127 | (36.1%) | 71 | (34.7%) | 96 | | (46.5%) | 206 | (53.1%) | <0.001 |  | 343 | (56.3%) | 714 | (42.5%) | 310 | (40.9%) | <0.001 |  | 243 | (32.8%) | 633 | | (54.4%) | 452 | | (43.3%) | <0.001 | |  |
| Neutral | 1337 | (32.4%) |  | 609 | (39.0%) | 118 | (31.6%) | 123 | (34.9%) | 58 | (28.2%) | 58 | | (28.4%) | 109 | (28.2%) |  |  | 185 | (30.4%) | 637 | (37.9%) | 245 | (32.3%) |  |  | 243 | (32.8%) | 370 | | (31.7%) | 419 | | (40.1%) |  | |  |
| More Willing | 1012 | (24.5%) |  | 222 | (14.2%) | 99 | (26.3%) | 102 | (29.0%) | 76 | (37.1%) | 51 | | (25.0%) | 73 | (18.7%) |  |  | 81 | (13.2%) | 329 | (19.6%) | 203 | (26.8%) |  |  | 256 | (34.5%) | 162 | | (13.9%) | 172 | | (16.5%) |  | |  |
| **How does this information [on the safety issue with the J&J vaccine] impact your trust of the safety monitoring system in place for vaccines? ^a^** | | | | | | | | | | | | | | | | | | | | | | | | | | | | | | | | | | | | | |
| Less Trust | 1785 | (26.0%) |  | 816 | (22.4%) | 164 | (24.1%) | 178 | (25.0%) | 99 | (25.6%) | 104 | | (31.7%) | 287 | (45.8%) | <0.001 |  | 358 | (34.2%) | 858 | (23.5%) | 406 | (25.2%) | <0.001 |  | 396 | (17.3%) | 664 | | (33.2%) | 513 | | (28.4%) | <0.001 | |  |
| Neutral | 2757 | (40.1%) |  | 1681 | (46.3%) | 262 | (38.5%) | 242 | (33.9%) | 121 | (31.1%) | 101 | | (30.8%) | 176 | (28.0%) |  |  | 395 | (37.8%) | 1583 | (43.4%) | 588 | (36.5%) |  |  | 882 | (38.5%) | 800 | | (39.9%) | 794 | | (43.9%) |  | |  |
| More Trust | 2325 | (33.9%) |  | 1138 | (31.3%) | 254 | (37.4%) | 292 | (41.0%) | 168 | (43.4%) | 123 | | (37.6%) | 164 | (26.1%) |  |  | 293 | (28.0%) | 1205 | (33.1%) | 619 | (38.4%) |  |  | 1015 | (44.3%) | 539 | | (26.9%) | 502 | | (27.7%) |  | |  |
| **How does this information on the safety issue with the J&J vaccine impact your feelings about your decision to get the vaccine? ^b^** | | | | | | | | | | | | | | | | | | | | | | | | | | | | | | | | | | | | | |
| Feel Worse | 179 | (5.7%) |  | 71 | (3.5%) | 15 | (5.2%) | 26 | (7.6%) | 18 | (10.7%) | 25 | | (25.0%) | 17 | (8.2%) | <0.001 |  | 23 | (5.6%) | 94 | (5.0%) | 52 | (6.5%) | 0.163 |  | 83 | (5.6%) | 52 | | (6.7%) | 33 | | (4.6%) | 0.008 | |  |
| Feel About the Same | 2011 | (63.6%) |  | 1384 | (68.9%) | 171 | (59.7%) | 182 | (53.2%) | 103 | (62.4%) | 40 | | (39.3%) | 107 | (53.0%) |  |  | 272 | (66.5%) | 1223 | (65.1%) | 480 | (60.5%) |  |  | 952 | (63.6%) | 467 | | (59.5%) | 489 | | (69.4%) |  | |  |
| Feel Better | 970 | (30.7%) |  | 553 | (27.6%) | 101 | (35.1%) | 134 | (39.2%) | 44 | (26.8%) | 36 | | (35.8%) | 78 | (38.7%) |  |  | 114 | (27.9%) | 561 | (29.9%) | 262 | (33.0%) |  |  | 461 | (30.8%) | 266 | | (33.9%) | 183 | | (26.0%) |  | |  |
| **When you were vaccinated, was any information about this safety issue with the J&J vaccine provided? ^b^** | | | | | | | | | | | | | | | | | | | | | | | | | | | | | | | | | | | | | |
| No discussion or written materials | 114 | (18.8%) |  | 44 | (19.0%) | 7 | (12.3%) | 10 | (14.9%) | 6 | (11.3%) | 5 | | (14.9%) | 38 | (27.6%) | 0.002 |  | 8 | (22.8%) | 26 | (19.8%) | 11 | (17.6%) | 0.981 |  | 12 | (14.6%) | 17 | | (22.4%) | 20 | | (28.3%) | 0.626 | |  |
| No discussion, but written materials | 74 | (12.2%) |  | 20 | (8.4%) | 10 | (18.3%) | 5 | (6.8%) | 7 | (13.0%) | 4 | | (13.3%) | 24 | (17.3%) |  |  | 4 | (10.6%) | 18 | (13.2%) | 8 | (12.8%) |  |  | 11 | (13.0%) | 9 | | (12.1%) | 9 | | (13.1%) |  | |  |
| Discussion, but no written materials | 115 | (18.8%) |  | 38 | (16.4%) | 8 | (15.4%) | 9 | (12.7%) | 10 | (19.8%) | 11 | | (32.7%) | 31 | (22.5%) |  |  | 5 | (15.3%) | 25 | (19.1%) | 9 | (15.1%) |  |  | 16 | (20.2%) | 14 | | (18.3%) | 9 | | (12.8%) |  | |  |
| Discussion and written materials | 305 | (50.2%) |  | 130 | (56.2%) | 30 | (54.2%) | 46 | (65.6%) | 29 | (55.9%) | 13 | | (39.1%) | 45 | (32.6%) |  |  | 18 | (51.4%) | 64 | (48.0%) | 33 | (54.5%) |  |  | 42 | (52.3%) | 35 | | (47.3%) | 33 | | (45.8%) |  | |  |
| **If you were going to get a COVID-19 vaccine and had a choice between the different vaccines, which vaccine would you get? ^c^** | | | | | | | | | | | | | | | | | | | | | | | | | | | | | | | | | | | | | |
| ***Johnson & Johnson*** |  |  |  |  |  |  |  |  |  |  |  |  | |  |  |  |  |  |  |  |  |  |  |  |  |  |  |  |  | |  |  | |  |  | |  |
| No | 2815 | (85.9%) |  | 1384 | (88.6%) | 313 | (83.6%) | 292 | (83.0%) | 160 | (78.0%) | 166 | | (81.0%) | 355 | (91.6%) | <0.001 |  | 552 | (90.6%) | 1430 | (85.1%) | 656 | (86.5%) | 0.005 |  | 615 | (82.9%) | 1019 | | (87.5%) | 919 | | (88.1%) | 0.005 | |  |
| Yes | 463 | (14.1%) |  | 178 | (11.4%) | 61 | (16.4%) | 60 | (17.0%) | 45 | (22.0%) | 39 | | (19.0%) | 33 | (8.4%) |  |  | 57 | (9.4%) | 250 | (14.9%) | 103 | (13.5%) |  |  | 127 | (17.1%) | 146 | | (12.5%) | 124 | | (11.9%) |  | |  |
| ***Moderna*** |  |  |  |  |  |  |  |  |  |  |  |  | |  |  |  |  |  |  |  |  |  |  |  |  |  |  |  |  | |  |  | |  |  | |  |
| No | 2712 | (82.8%) |  | 1333 | (85.4%) | 297 | (79.3%) | 272 | (77.3%) | 161 | (78.4%) | 158 | | (76.9%) | 349 | (90.1%) | <0.001 |  | 524 | (86.0%) | 1390 | (82.7%) | 627 | (82.6%) | 0.183 |  | 546 | (73.6%) | 1025 | | (88.0%) | 886 | | (84.9%) | <0.001 | |  |
| Yes | 565 | (17.2%) |  | 229 | (14.6%) | 77 | (20.7%) | 80 | (22.7%) | 44 | (21.6%) | 47 | | (23.1%) | 39 | (9.9%) |  |  | 86 | (14.0%) | 291 | (17.3%) | 132 | (17.4%) |  |  | 196 | (26.4%) | 140 | | (12.0%) | 158 | | (15.1%) |  | |  |
| ***Pfizer*** |  |  |  |  |  |  |  |  |  |  |  |  | |  |  |  |  |  |  |  |  |  |  |  |  |  |  |  |  | |  |  | |  |  | |  |
| No | 2577 | (78.6%) |  | 1280 | (82.0%) | 272 | (72.7%) | 260 | (73.7%) | 142 | (69.2%) | 152 | | (74.0%) | 330 | (85.3%) | <0.001 |  | 539 | (88.4%) | 1288 | (76.6%) | 580 | (76.4%) | <0.001 |  | 498 | (67.1%) | 992 | | (85.1%) | 845 | | (80.9%) | <0.001 | |  |
| Yes | 701 | (21.4%) |  | 282 | (18.0%) | 102 | (27.3%) | 93 | (26.3%) | 63 | (30.8%) | 54 | | (26.0%) | 57 | (14.7%) |  |  | 70 | (11.6%) | 393 | (23.4%) | 179 | (23.6%) |  |  | 244 | (32.9%) | 174 | | (14.9%) | 199 | | (19.1%) |  | |  |
| ***Whichever Vaccine Offered*** | | | | | | | | | | | | | | | | | | | | | | | | | | | | | | | | | | | | | |
| No | 2753 | (84.0%) |  | 1348 | (86.3%) | 302 | (80.5%) | 280 | (79.3%) | 164 | (79.7%) | 170 | | (82.7%) | 331 | (85.5%) | 0.003 |  | 535 | (87.9%) | 1397 | (83.1%) | 628 | (82.8%) | 0.020 |  | 593 | (79.9%) | 1039 | | (89.1%) | 850 | | (81.4%) | <0.001 | |  |
| Yes | 524 | (16.0%) |  | 214 | (13.7%) | 73 | (19.5%) | 73 | (20.7%) | 42 | (20.3%) | 36 | | (17.3%) | 56 | (14.5%) |  |  | 74 | (12.1%) | 284 | (16.9%) | 131 | (17.2%) |  |  | 149 | (20.1%) | 127 | | (10.9%) | 194 | | (18.6%) |  | |  |
| ***Would Not Get Vaccinated*** | | | | | | | | | | | | | | | | | | | | | | | | | | | | | | | | | | | | | |
| No | 1813 | (55.3%) |  | 734 | (47.0%) | 239 | (63.8%) | 249 | (70.6%) | 152 | (73.8%) | 133 | | (64.9%) | 159 | (41.0%) | <0.001 |  | 233 | (38.3%) | 973 | (57.9%) | 438 | (57.7%) | <0.001 |  | 546 | (73.7%) | 480 | | (41.2%) | 560 | | (53.6%) | <0.001 | |  |
| Yes | 1464 | (44.7%) |  | 828 | (53.0%) | 136 | (36.2%) | 104 | (29.4%) | 54 | (26.2%) | 72 | | (35.1%) | 229 | (59.0%) |  |  | 376 | (61.7%) | 707 | (42.1%) | 321 | (42.3%) |  |  | 195 | (26.3%) | 686 | | (58.8%) | 484 | | (46.4%) |  | |  |
| **If the J&J vaccine was the only COVID-19 vaccine you could get in the next month, would you get it? ^c^** | | | | | | | | | | | | | | | | | | | | | | | | | | | | | | | | | | | | | |
| No | 2162 | (68.7%) |  | 1157 | (74.1%) | 257 | (68.5%) | 199 | (56.3%) | 104 | (50.6%) | 119 | | (58.0%) | 289 | (74.6%) | <0.001 |  | 465 | (76.4%) | 1131 | (67.3%) | 501 | (66.0%) | <0.001 |  | 415 | (56.0%) | 875 | | (75.0%) | 735 | | (70.5%) | <0.001 | |  |
| Yes | 984 | (31.3%) |  | 405 | (25.9%) | 118 | (31.5%) | 154 | (43.7%) | 102 | (49.4%) | 86 | | (42.0%) | 98 | (25.4%) |  |  | 144 | (23.6%) | 549 | (32.7%) | 258 | (34.0%) |  |  | 326 | (44.0%) | 291 | | (25.0%) | 308 | | (29.5%) |  | |  |
| **How much do you trust the Centers for Disease Control and Prevention (CDC)? ^a^** | | | | | | | | | | | | | | | | | | | | | | | | | | | | | | | | | | | | | |
| Distrust/Neutral | 14410 | (57.8%) |  | 1708 | (47.0%) | 325 | (47.8%) | 333 | (46.7%) | 191 | (49.4%) | 214 | | (65.5%) | 435 | (69.4%) | <0.001 |  | 639 | (61.0%) | 1812 | (49.7%) | 721 | (44.7%) | <0.001 |  | 667 | (29.1%) | 1345 | | (67.2%) | 1065 | | (58.9%) | <0.001 | |  |
| Trust | 10515 | (42.2%) |  | 1927 | (53.0%) | 356 | (52.2%) | 379 | (53.3%) | 196 | (50.6%) | 113 | | (34.5%) | 192 | (30.6%) |  |  | 408 | (39.0%) | 1835 | (50.3%) | 892 | (55.3%) |  |  | 1627 | (70.9%) | 657 | | (32.8%) | 744 | | (41.1%) |  | |  |
| **How much do you trust the Food and Drug Administration (FDA)? ^a^** | | | | | | | | | | | | | | | | | | | | | | | | | | | | | | | | | | | | | |
| Distrust/Neutral | 12929 | (57.9%) |  | 1766 | (48.6%) | 316 | (46.4%) | 327 | (45.9%) | 202 | (52.2%) | 201 | | (61.4%) | 447 | (71.2%) | <0.001 |  | 634 | (60.6%) | 1848 | (50.7%) | 745 | (46.2%) | <0.001 |  | 740 | (32.3%) | 1314 | | (65.6%) | 1078 | | (59.6%) | <0.001 | |  |
| Trust | 9398 | (42.1%) |  | 1869 | (51.4%) | 365 | (53.6%) | 385 | (54.1%) | 186 | (47.8%) | 126 | | (38.6%) | 180 | (28.8%) |  |  | 413 | (39.4%) | 1798 | (49.3%) | 867 | (53.8%) |  |  | 1553 | (67.7%) | 689 | | (34.4%) | 732 | | (40.4%) |  | |  |
