# Supplementary material for: Impact of vaccine pause due to Thrombosis with thrombocytopenia syndrome (TTS) following vaccination with the Ad26.COV2.S vaccine manufactured by Janssen/Johnson & Johnson on vaccine hesitancy and acceptance among the unvaccinated population
Source: PLoS One. 2022 Oct 11;17(10):e0274443. doi: 10.1371/journal.pone.0274443 (PMC9553048; doi:10.1371/journal.pone.0274443)
Supplement: S1 Survey — (DOCX) [file pone.0274443.s006.docx]

**Survey 1 (Vaccination Intent): Survey Questions and Skip/Display Logic**

*Note. Skip/display logic specified in italics*

What is your age?

What is your gender?

Male

Female

Have you had COVID-19?

Yes, I was diagnosed by my doctor or other provider (nurse, physician assistant)

Yes, I tested positive for COVID-19 but didn't see a doctor

Unsure, I think I had symptoms of COVID-19 but did not get a test

No

How do you most strongly identify?

White

African American or Black

Hispanic / LatinX

Asian

Native American or American Indian

Alaskan Native

Other ethnic group

*For those who chose “Hispanic/LatinX” or “Asian”*

What country or region of origin is your family from?

*For those who chose “Hispanic/LatinX”*

Caribbean

South America

Central America

Europe

Mexico

*For those who chose “Asian”*

Bangladesh

China

India

Japan

Nepal

Pakistan

Philippines

South Korea

Vietnam

*For those who chose “Hispanic/LatinX” or “Asian”*

Other

What will you do when it is your turn to get the COVID-19 vaccine, at no cost?

I have already received at least one dose of the vaccine

I will definitely get it as soon as I can

I will likely get it as soon as I can

I will likely get it but not right away

I will likely not get vaccinated

I will definitely not get vaccinated

*For those who did* ***not*** *indicate that they would “definitely not get vaccinated” (diminishing list of response options)*

Of the below, what would be your biggest reason to get the COVID-19 vaccine?

Of the remaining, what would be your second biggest reason to get the COVID-19 vaccine?

Of the remaining, what would be your third biggest reason to get the COVID-19 vaccine?

Keep myself from getting very sick or hospitalized

Protect my family members

Protect vulnerable people in the community

Start to return to a more normal life

Everyone else is getting vaccinated

My doctor says I should

It is encouraged by my work

*For those who did* ***not*** *indicate that they were already vaccinated or would “definitely get it as soon as I can” (question wording specific to intention response)*

You said you would likely get it as soon as you can. Why not definitely? Select up to 3

What are your main reasons for not wanting to get the COVID-19 vaccine right away? Select up to 3

What are your main reasons for likely not wanting to get the COVID-19 vaccine? Select up to 3

What are your main reasons for definitely not wanting to get the COVID-19 vaccine? Select up to 3

I distrust the medical system because it has mistreated people like me

There were not enough people like me who were a part of the vaccine studies

I don't think I am at risk of getting COVID-19

If I got COVID-19 I don't think I would get very sick

I don't think the COVID-19 vaccines are likely to protect me from COVID-19 or the variants

I am worried about the safety of COVID-19 vaccines

I'm worried about having to provide personal information to get the vaccine

It is difficult or inconvenient for me to go get the vaccine

I have a health condition that may put me at increased risk for COVID-19 vaccine side effects

I am waiting for herd immunity (enough people gain immunity that I am not exposed)

I prefer natural immunity by recovering from COVID-19 myself

It is against my religious beliefs

Other reason

No more, continue

*For those who chose “other reason”*

What is your other reason for not wanting to get the vaccine?

[open text answer box]

How much do you trust the below to help you decide on whether or not to receive the COVID-19 vaccine?

*Targets (target presentation randomized—6 of 13 targets displayed)*

My doctor or other healthcare provider

My local or state health department

Health officials from the Centers for Disease Control and Prevention (CDC)

The Surgeon General

Scientists and doctors from Universities

Dr. Anthony Fauci

My religious leader

Somebody I know who works in healthcare

News commentators

Mayor, governor or other elected official

Entertainers

Sports figures

My family and close friends

*Response option*

1 - Highly distrust

2

3 - Neutral

4

5 - Highly trust

Are you concerned at all about the safety of the COVID-19 vaccines?

Very concerned

Concerned

A little concerned

Not at all concerned

*For those who did* ***not*** *indicate that they were “not at all concerned”*

Which of the following are you most concerned about for the COVID-19 vaccine? Select up to 3.

The COVID-19 vaccines were developed too fast

A lot of people who get the vaccine feel tired, achy and get headaches and fever the next day

Some people have bad allergic reactions to COVID-19 vaccines

I am not sure the ingredients in COVID-19 vaccines are safe

I hear that COVID-19 vaccines might change my DNA

I hear that COVID-19 vaccines might affect fertility or ability to have children

I hear that they use aborted fetuses in making the COVID-19 vaccine

I hear that COVID-19 vaccines are hurting or killing elderly people

There may be side effects to COVID-19 that haven't been figured out yet

Drug companies are experimenting on people with the COVID-19 vaccine

The drug companies just care about making a lot of money from COVID-19 vaccines

Other

No more, continue

*For those who chose “other”*

What other safety concerns do you have about the COVID-19 vaccine?

[open-text]

Has a close friend or family member been ill with COVID-19?

Yes

No

*For those who chose “yes”*

Of your close friends or family, what was the most severe experience?

Not hospitalized, fully recovered with no continued COVID-19 related health problems

Not hospitalized, partially recovered but with continued COVID-19 related health problems

Hospitalized, fully recovered with no continued COVID-19 related health problems

Hospitalized, partially recovered but with continued COVID-19 related health problems

Passed away

When thinking of the people you frequently interact with, what do you think are their most common reasons for not wanting to get the vaccine? Select up to 3

They distrust the medical system because it has mistreated people like them

There were not enough people like them who were a part of the vaccine studies

They don't think they are at risk of getting COVID-19

If they got COVID-19 they don't think they would get very sick

They don’t think the COVID-19 vaccines are likely to protect them from COVID-19 or the variants

They are worried about the safety of COVID-19 vaccines

They are worried about having to provide personal information to get the vaccine

It is difficult or inconvenient for them to go get the vaccine

They have health conditions that may put them at increased risk for COVID-19 vaccine side effects

They are waiting for herd immunity (enough people gain immunity that they are not exposed)

They prefer natural immunity by recovering from COVID-19 themselves

It is against their religious beliefs

Other reason

No more continue

*For those who chose “other reason”*

Why else do you think the people you interact with do not want to get the vaccine?

[open text box]

How important do you think a COVID-19 vaccine is to stop the spread of infection in the US?

Very important

Important

Unimportant

Very unimportant

How likely are you to discuss the COVID-19 vaccine with your healthcare provider?

Very likely

Likely

Unlikely

Very unlikely

During COVID-19 I have no access to my healthcare provider

I don't have a regular healthcare provider

If you become infected with COVID-19, how severe do you think the infection will be?

Very severe – may cause death

Somewhat severe – may cause hospitalization

Not very severe – may cause a few days or week of being in bed at home

Mild – able to still carry-on activities while at home

I will not get sick at all

*For those who did indicated that their reaction would be “very severe” or “somewhat severe”*

Why do you believe your infection would be severe? Select any that apply

I have a medical condition

I have difficulty accessing health care or treatment

From what I see in the news about COVID-19

I know people who have had COVID-19 and they were very sick

My age

Other

No more, continue

*For those who chose “other”*

What other reason makes you believe your infection would be severe?

[open-text]

*For those who did indicated that their reaction would be “not very severe,” “mild,” or that they would “not get sick at all” (question wording specific to severity response)*

Why do you think the infection would be mild or not very severe? Select any that apply

Why do you think you will not get sick at all? Select any that apply

I am a healthy person

I have easy access to quality healthcare

I think the COVID-19 pandemic is blown out of proportion

I know people who have had COVID-19 and they were not very sick

My age

Other

No more, continue

*For those who chose “other”*

What other reason?

[open-text]

Which of the statements below most applies to you?

I came here from another country

At least one of my parents came here from another country

At least one of my grandparents came here from another country

None of the above apply

Which religious faith do you most strongly identify with?

Buddhism

Christianity

Hinduism

Islam

Judaism

Other

No religion

How would you describe the area in which you live?

Large city

Suburb

Town / village

Rural area / farm

What is the highest level of education you have completed?

High School degree or less

Technical or vocational training

College Degree

Masters degree or higher

Approximately how much is your annual household income? (Before taxes)

Under $20,000

$20,000 - $50,000

$50,001 - $75,000

$75,001 - $125,000

$125,001 - $250,000

Over $250,000

In general, do you think of yourself politically as…

Democrat

Independent

Republican
