# Supplementary material for: Impact of vaccine pause due to Thrombosis with thrombocytopenia syndrome (TTS) following vaccination with the Ad26.COV2.S vaccine manufactured by Janssen/Johnson & Johnson on vaccine hesitancy and acceptance among the unvaccinated population
Source: PLoS One. 2022 Oct 11;17(10):e0274443. doi: 10.1371/journal.pone.0274443 (PMC9553048; doi:10.1371/journal.pone.0274443)
Supplement: S2 Survey — (DOCX) [file pone.0274443.s007.docx]

**Survey 2 (Rapid Response): Survey Questions and Skip/Display Logic**

*Note. Skip/display logic specified in italics*

What is your age?

What is your gender?

Male

Female

What will you do when it is your turn to get the COVID-19 vaccine, at no cost?

I have already received at least one dose of the vaccine

I will definitely get it as soon as I can

I will likely get it as soon as I can

I will likely get it but not right away

I will likely not get vaccinated

I will definitely not get vaccinated

*For those who indicated they have been vaccinated*

Which vaccine did you receive?

Pfizer

Moderna

Johnson & Johnson

I don't remember

*For those who indicated they have been vaccinated*

Did you choose which vaccine to receive?

Yes

No

*For those who indicated they had a choice of vaccine*

Which vaccines were available for you to choose from? [select all that apply]

Pfizer

Moderna

Johnson & Johnson

I don't remember

*For those who indicated they have been vaccinated*

About when did you receive your first COVID-19 vaccination? Month:

December 2020

January 2021

February 2021

March 2021

April 2021

May 2021

*For those who indicated they have been vaccinated*

About when did you receive your first vaccination? Day:

[number of days in the respective month]

How much do you trust the Centers for Disease Control and Prevention (CDC)?

Trust Very Much

Trust Somewhat

Neutral

Distrust Somewhat

Distrust Very Much

How much do you trust the Food and Drug Administration (FDA)?

Trust Very Much

Trust Somewhat

Neutral

Distrust Somewhat

Distrust Very Much

Have you heard about any safety issues with the Johnson & Johnson (J&J) vaccine?

Yes

No

*For those who indicated they have heard about safety issues with J&J*

What have you heard?

[open text, not forced]

*For those who indicated they have heard about safety issues with J&J and have* ***not*** *been vaccinated*

How has what you've heard impacted your willingness to get the J&J vaccine?

I am now much more willing

I am now somewhat more willing

The pause did not impact my willingness

I am now somewhat less willing

I am now much less willing

*For those who indicated they have heard about safety issues with J&J and have* ***not*** *been vaccinated*

How has what you've heard impacted your willingness to get a different COVID-19 vaccine (Pfizer or Moderna)?

I am now much more willing

I am now somewhat more willing

The pause did not impact my willingness

I am now somewhat less willing

I am now much less willing

*For those who indicated they have heard about safety issues with J&J*

How has what you've heard impacted your trust of the safety monitoring system in place for vaccines?

I have much more trust in the vaccine safety system

I have somewhat more trust in the vaccine safety system

It hasn't changed my trust in the vaccine safety system

I have somewhat less trust in the vaccine safety system

I have much less trust in the vaccine safety system

Here is a quick summary of a recent safety issue with the J&J vaccine based on language from the CDC website: On April 13, the CDC and FDA paused the use of the J&J vaccine in the US after reports of six people developing a rare and severe type of blood clot after getting the J&J vaccine. During the pause, the FDA and CDC conducted a thorough review of the available data. They determined that the vaccine’s benefits still outweigh its risks, as the chance of these clots occurring is very low. The FDA and CDC also reached out to healthcare providers to ensure they were aware of and could manage these potential adverse events. On April 25, the CDC and FDA lifted the pause and use of the J&J vaccine resumed. The J&J vaccine has advantages over the other COVID-19 vaccines, including only needing one shot instead of two.

Continue

*For those who indicated they have* ***not*** *been vaccinated*

How does this information impact your willingness to get the J&J vaccine?

I am now much more willing

I am now somewhat more willing

The pause did not impact my willingness

I am now somewhat less willing

I am now much less willing

*For those who indicated they have* ***not*** *been vaccinated*

How does this information impact your willingness to get a different COVID-19 vaccine (Pfizer or Moderna)?

I am now much more willing

I am now somewhat more willing

The pause did not impact my willingness

I am now somewhat less willing

I am now much less willing

How does this information impact your trust of the safety monitoring system in place for vaccines?

I have much more trust in the vaccine safety system

I have somewhat more trust in the vaccine safety system

It hasn't changed my trust in the vaccine safety system

I have somewhat less trust in the vaccine safety system

I have much less trust in the vaccine safety system

*For those who indicated they have been vaccinated and received J&J*

How does this information on the safety issue with the J&J vaccine impact your feelings about your decision to get the J&J vaccine?

I feel worse about it

I feel the same about it

I feel better about it

*For those who indicated they have been vaccinated and received J&J* ***on or after April 24^th^***

When you were vaccinated, was any information about this safety issue with the J&J vaccine provided?

Yes, someone discussed it with me, and I received written materials describing it

Yes, someone discussed it with me, but I did not receive any written materials describing it

No one discussed it with me but I did receive a fact sheet that mentioned it

No one discussed it with me, and I did not receive any materials mentioning it

*For those who indicated they have been vaccinated and received Moderna*

How does this information about the safety issue with the J&J vaccine impact your feelings about your decision to get the Moderna vaccine?

I feel worse about it

I feel the same about it

I feel better about it

*For those who indicated they have been vaccinated and received Pfizer*

How does this information about the safety issue with the J&J vaccine impact your feelings about your decision to get the Pfizer vaccine?

I feel worse about it

I feel the same about it

I feel better about it

*For those who indicated they have* ***not*** *been vaccinated*

If you were going to get a COVID-19 vaccine and had a choice between the different vaccines, which vaccine would you get? Select all that apply

Pfizer vaccine

Moderna vaccine

Johnson & Johnson vaccine

Whichever vaccine was offered to me

I would not get any vaccine

*For those who indicated they have* ***not*** *been vaccinated*

If the J&J vaccine was the only COVID-19 vaccine you could get in the next month, would you get it?

Yes

No

How do you most strongly identify?

White

African American or Black

Hispanic / LatinX

Asian

Native American or American Indian

Alaskan Native

Other ethnic group

How would you describe the area in which you live?

Large city

Suburb

Town / village

Rural area / farm

In general, do you think of yourself politically as…

Democrat

Independent but I lean Democrat

Independent

Independent but I lean Republican

Republican

What is the highest level of education you have completed?

High School degree or less

Technical or vocational training

College Degree

Masters degree or higher

Approximately how much is your annual household income? (Before taxes)

Under $20,000

$20,000 - $50,000

$50,001 - $75,000

$75,001 - $125,000

$125,001 - $250,000

Over $250,000
